# Single-stage revision for chronic periprosthetic joint infection after knee and hip arthroplasties: indications and treatments

**DOI:** 10.1186/s42836-023-00168-5

**Published:** 2023-03-02

**Authors:** Wenbo Mu, Baochao Ji, Li Cao

**Affiliations:** 1 Department of Orthopedics, the First Affiliated Hospital of Xinjiang Medical University, Urumqi, 830011 Xinjiang China; 2 Department of Pharmacognosy, School of Pharmacy, Xinjiang Medical University, Urumqi, 830011 Xinjiang China

**Keywords:** Single-stage, Chronic, Periprosthetic joint infection, Indication

## Abstract

Single-stage revision for chronic periprosthetic joint infection has been introduced 40 years ago. This option is gaining more and more attention as well as popularity. It is a reliable treatment for the chronic periprosthetic joint infection after knee and hip arthroplasties when implemented by an experienced multi-disciplinary team. However, its indications and corresponding treatments remain controversial. This review focused on the indications and specific treatments related to the option, with an attempt to help surgeons to use this method with more favorable outcomes.

## Introduction

Periprosthetic joint infection (PJI) remains the most devastating complication following total hip arthroplasty (THA) and total knee arthroplasty (TKA). Though the occurrence of PJI reportedly stood somewhere at 0.5–2% following primary arthroplasty and at 3–10% after revision arthroplasty, periprosthetic joint infection is the leading cause for revision after total knee arthroplasty and ranks No. 3 for revision total hip arthroplasty [1]. It not only results in longer patient immobilization, and extended hospitalization, causing physical and mental morbidity, but also imposes a remarkable healthcare burden, which was reported to be $100,000 per episode and exceed $1.62 billion in 2020 [2]. The PJI treatment is designed to alleviate symptoms and preserve functions of affected limbs while achieving patient satisfaction and cost-effectiveness.

The optimal surgical treatment for PJI remains controversial and the current treatment strategy is based on several factors, such as symptom duration, pathogenic microorganisms, host status, among others*.* The most widely employed management include debridement, antibiotic use and implant retention (DAIR), single- or two-stage revision whereas arthrodesis and amputation are less popular. Carlsson *et al.,* for the first time, reported a single-stage revision after hip arthroplasty 44 years ago [3, 4]. This treatment strategy allows surgeons to achieve implant removal, thorough debridement of infected sources in the joint cavity and new prosthetic implantation with one-time surgery. Single-stage revision is a patient-centered solution as it potentially has a wide array of advantages, including shorter hospitalization, less anesthetic risk, morbidity, and mortality, lower rates of complications, such as dislocation, periprosthetic fracture, earlier return to activity, better limb function and higher patient satisfaction rate as well as lower socioeconomic burden [5–8]. It's appealing to both surgeons and patients and gaining popularity worldwide with infection control rate non-inferior to two-stage revision [9–11]. Nonetheless, single-stage revision should not be performed until a versatile team and specific protocols are well-established. The practice of single-stage revision should involve a multi-disciplinary team operating in both proactive and reactive manners. As to the surgeons, this management plan should not be carried out unless they are skilled in the following treatments: debridement, antibiotic use, and implant retention with modular components exchanged, two-stage revision, arthrodesis, amputation as well as antibiotic suppression.

### Indications and contraindications

It's not possible to deal with potential PJI scenarios with a "one-for-all" protocol. One-stage revision should also be performed in selected patients. Indications and contraindications for single-stage revision vary with different institutions or settings, which are constantly evolving (Table 1). Some surgeons stick to established indications while others are more open-minded. In our setting, we routinely perform single-stage revision on broader indications while accomplishing satisfactory infection control rate [12–14].

**Table 1:** Indications and contraindications for single-stage revision

Institutes	Indications	Contraindications
First Affiliated Hospital of Xinjiang Medical University, Urumqi [15]	Good soft tissue No active systemic sepsis Non-severely immuno-compromised host	Active systemic infection Infection involving neurovascular bundles and peripheral vascular disease
Universitätsmedizin Berlin [16]	Good soft tissue No massive bone loss Prior revisions less than 2 times	Difficult-to-treat pathogens
Endo-Klinik Hamburg [17]	Known pathogen Pathogen susceptible to antibiotics	Prior failed revisions more than 2 times Infection involving neurovascular bundles Sinus tract with unidentified pathogen
University College of London Hospital [18]	Known pathogen Pathogen susceptible to antibiotics Good soft tissue	Significant bone loss Peripheral vascular disease Immuno-compromised host Concurrent sepsis Systemic disease Polymicrobial infection
2018 International Consensus Meeting [19]	Known pathogen Pathogen susceptible to antibiotics Good soft tissue Absence of massive bone loss	Immuno-compromised host Active systemic infection Radical debridement not possible Local antimicrobial treatment not possible
Infectious Disease Society of America [20]	Known pathogen Pathogen susceptible to antibiotics with oral bioavailability Good soft tissue No massive bone loss No bone graft needed Antibiotic-loaded bone cement applied for fixation	No prior two-stage revision

### Patient positioning

Aspiration for the infected joint is routinely conducted prior to revision surgery for microbiological tests. For patients undergoing hip revision, lateral decubitus position is applied with the pelvis fixed well. Cushion is placed between the legs, with the operated extremity able to move freely. For patients receiving revision knee arthroplasty, supine position is assumed with the knee joint able to flex to 110°. A tourniquet is routinely placed on the proximal thigh and inflated without using esmarch bandage. Tranexamic acid is routinely administrated intravenously prior to incision.

### Surgical approach

A posterolateral approach is routinely adopted in revision hip surgery while midline incision is made and medial parapatellar approach is adopted in revision knee surgery. Good exposure is the first requirement and may be difficult to achieve in revision surgery. Therefore, the incision made should be large enough. The previous skin scar and the sinus tract should be excised. The joint fluid is collected intraoperatively and further analyzed to identify the microorganism(s).

### Debridement

Radical debridement is crucial to achieve infection eradication. It includes mechanical debridement and chemical debridement.

Aggressive mechanical debridement involves the removal of all suspected bone sequela, necrotic and fibrous tissues, non-absorbable sutures as well as proliferative inflammatory synovium in the surgical field. A surgical boundary with bleeding, fresh soft tissue is vital for radical debridement. This is accomplished with the usage of large and small curettes, rougeurs, surgical knife and electrotome. The knee joint requires radical compartmental debridement, including the medial gutter, lateral gutter, suprapatellar pouch and the posterior capsule area. Cautions should be exercised when debriding the collateral ligament. Multiple samples (more than 5) should be taken from the highly-suspected infected area, such as pseudocapsules and sent for culture, antibiotic sensitivity test and histological examination. The position of the prosthesis is evaluated and the prosthesis is checked to see if it's solidly fixed or not with manual compression as well as instrumented probing. A scalpel is used to try to penetrate the prosthesis-bone interface. An attempt to pull out the prosthesis with extractor is made even if a scalpel can not be inserted into the interface. The prosthesis is considered fixed solidly if it remains *in situ* [21]. Sterile stiff brush is utilized to remove the biofilm from the surface of the in-site prosthesis. If the position of the prosthesis is not satisfactory or the fixation is considered not solid. The prosthesis, cement and remaining implant such wires are then taken out with specific explant device to conserve native bone as much as possible. The posterior knee joint is exposed for debridement. Intramedullary surface is debrided with burr and curette to remove the membrane, avascular neo-cortex, inflammatory tissues, biofilm and occlusive bony shelf until fresh bleeding bony bed is obtained.

It is rarely possible to fully eradicate infectious tissues with mechanical debridement. Chemical debridement is applied subsequently to create a clean surgical field hostile to microorganisms. It was reported that hydrogen peroxide of 3% could inhibit biofilm formation through broad antimicrobial ability and direct bactericidal effect. Povidone-iodine consists of polyvinylpyrrolidone and 1% iodine [22]. Bacteriostatic effect was demonstrated when hydrogen peroxide of 3% and povidone-iodine were used separately, whereas they were of bactericidal nature when applied in combination [23].

The surgical field is irrigated with normal saline, 0.5% aqueous povidone-iodine, normal saline, 3% hydrogen peroxide and normal saline sequentially. The 0.5% aqueous povidone-iodine should be left to stay in the wound for at least 5 min instead of being washed out immediately, which allows for adequate time for the povidone-iodine to fully exert its antimicrobial effect [24]. Gentle washing with manual syringes instead of pulsative lavage was used in this step. This is to avoid iatrogenic dispersal of microorganisms into deeper space. Any remaining necrotic tissue or cement should be inspected and taken out. The surgical field is immersed with 0.5% aqueous povidone-iodine and gauze is placed onto the wound. The wound is temporarily closed with interrupted sutures for at least 15 min.

### Intermission

The previously used drapes are removed and the all surgeons of the surgical team take off and discard their used gowns and gloves. Used surgical sets, suction catheter, electrotome and light handles are removed from theater. The patient is approached again in the way at the start of the surgery. All the surgeons wear new gowns and gloves. New sterile surgical instruments are brought into the theater for re-implantation. Separate dual surgical setup has been shown to increase infection control rate significantly compared with single setup in PJI cases [25]. The wound is then opened, with sutures and gauze discarded. The entire surgical field undergoes another round of lavage. Instead of washing with manual syringes, pulsative lavage is used at this stage to achieve tidal effect. The acetabular cavity, femoral canal and tibial canal are lavaged to wash away any potential residual micro-debris and membrane. A total of at least 6 L fluid is applied in the chemical debridement.

### Reconstruction

The bony bed is prepared and bone stock and stability are further evaluated. Vancomycin powder of 1 g is poured into the acetabulum and femoral canal in hip revision surgery while into distal femoral canal and proximal tibial canal in knee revision surgery. A new prosthesis is then implanted. Substantial controversy remains regarding the way of fixation. Historically, cemented re-implantation has been advocated since polymethylmethacrylate (PMMA) acts as a carrier for local antibiotic elution, which is considered critical to the success. Antibiotics were thought to elute from the pores in the cement and follow, in a concentration gradient, into the surrounding tissues and bone. However, the antibiotics powder does not distribute into the cement evenly and only those entrapped at the cement surface dissolve into the surrounding tissues. Besides, it has been shown that the antibiotics released from the PMMA are merely about 1–15% [26]. Moreover, the cement is mechanically compromised and long-term stability may suffer [27]. Meanwhile, excellent results were reported from surgeons using cementless implants in septic revisions [28–30]. The logic that cemented fixation is mandatory for topical antibiotic delivery should be re-visited. In our practice, prosthesis with cementless fixation is the first priority in hip revision surgery and cemented fixation is applied in all knee revision surgeries. Vancomycin powder is mixed with cement if bone graft is needed and commercial cement impregnated with 0.5 g gentamycin is used (Zimmer Biomet Orthopaedics, Winterthur, Switzerland). A drainage is routinely placed distally in the hip and laterally in the knee. Besides, a t-branch pipe is routinely installed proximally in the hip for topical antibiotic infusion postoperatively. Antibiotics powder of 0.5 g is poured into the joint cavity prior to deep fascia closure. Tranexamic acid of 1 g is injected into the joint cavity.

### Antibiotics

Proper antibiotics are administrated since it is critical to the success of infection eradication. The antibiotic protocol, worked out by the multidisciplinary team (MDT), included the identification of microorganisms, dosage, duration, duality and delivery, and is designated as 5D for short [31].

Our antibiotic treatment protocol is composed of intravenous, intra-articular infusion and oral delivery on the basis of the patients' weight and culture results (Table 2).

**Table 2:** Antibiotic treatment protocol [21]

	**Positive Bacterium culture**	**Negative Bacterium culture**
Intravenous Preoperative	Pathogen-sensitive antibiotics	Vancomycin of 1 g
Topical Intraoperative	Vancomycin powder of 0.5 g/Meropenem powder of 0.5 g	Vancomycin powder of 0.5 g
Intravenous Postoperative	Pathogen-sensitive antibiotics for 2 w	Vancomycin every 12 h for 2 w
Intra-articular Infusion Postoperative	Multi-drug-resistant bacteria, Fungi, polymicrobial infection: pathogen-sensitive antibiotics for 12–14 d	Vancomycin of 0.5 g in the morning and Meropenem of 0.5 g in the afternoon for 12–14 d
Oral Postoperative	After the intravenous and topical antibiotic administration, Quinolones with Rifampicin, as oral switch therapy, for at least 31 d until erythrocyte sedimentation rate (ESR) and C-reactive protein continue to decrease to normal range or remain stable and are close to the normal level.	

Since the biofilm remnant may remain in the joint even after the thorough debridement, antibiotics that can suppress biofilm should be applied afterward. It has been shown that if pathogens reside in the biofilm, the minimum biofilm eradication concentrations (MBECS) of antibiotics needed is about a hundred to a thousand times over the minimum inhibitory concentrations (MIC) required to eradicate the planktonic microorganism. Intravenous delivery of antimicrobial agents would cause systemic toxicity before reaching MBECs at the site of joint [32]. This makes topical antibiotic delivery crucial. It can be achieved in the form of antibiotic beads, antibiotic sponges as well as antibiotic powder. Nevertheless, it has not been reported whether antibiotic beads outperform antibiotic powder in treating chronic PJI or *vice versa*.

It has been proved that antibiotic-impregnated PMMA is not released in a constant manner for a sufficiently long time. It has been shown that the majority of the antibiotics elution occurred within the first 48–72 h and the local antibiotic concentration dropped to a sub-therapeutic level afterward. Besides, additional surgery is required to remove it owing to its non-biodegradable feature. Moreover, bacteria can adhere to the surface of PMMA beads that are left in the joint, resulting in new biofilm formation. PMMA beads might also act as a third body to scratch the man-made joint surfaces. In addition, only the antibiotics that remain stable under heat can be used with PMMA [33]. Investigations on calcium sulphate beads with bioabsorbability are underway to address these issues. Nevertheless, wound drainage, heterotopic ossification along with hypercalcemia have been reported as related complications [34]. We use antibiotic powder since it is economic-friendly and easily available. Multiple studies have proved its efficacy in providing high local antibiotic concentrations whilst avoiding local and systemic complications [35, 36].

In our practice of intra-articular antibiotic infusion. The drainage was shut prior to intra-articular antibiotics delivery and kept shut for 20 h afterwards until 3 h prior to next delivery. The topical antibiotics are injected into the joint after the joint fluid is extracted with the t-branch pipe for hip or with syringe for knee in a sterile fashion. The drainage is taken away if draining fluid volume is less than 50 mL. The t-branch pipe was taken away if the intra-articular antibiotic infusion ceases.

## Conclusions

Single-stage revision is a reliable strategy for treating chronic PJI with the synergy of experienced MDT. The success relies on proper patient selection, adequate exposure, thorough reproducible debridement, sophisticated reconstruction technique, appropriate antibiotic protocol as well as follow-up.

## Data Availability

Not applicable.
